# Why do people move? Enhancing human mobility prediction using local functions based on public records and SNS data

**DOI:** 10.1371/journal.pone.0192698

**Published:** 2018-02-12

**Authors:** Jungmin Kim, Juyong Park, Wonjae Lee

**Affiliations:** Graduate School of Culture Technology, Korea Advanced Institute of Science and Technology, Daejeon, South Korea; Dalian University of Technology, CHINA

## Abstract

The quality of life for people in urban regions can be improved by predicting urban human mobility and adjusting urban planning accordingly. In this study, we compared several possible variables to verify whether a gravity model (a human mobility prediction model borrowed from Newtonian mechanics) worked as well in inner-city regions as it did in intra-city regions. We reviewed the resident population, the number of employees, and the number of SNS posts as variables for generating mass values for an urban traffic gravity model. We also compared the straight-line distance, travel distance, and the impact of time as possible distance values. We defined the functions of urban regions on the basis of public records and SNS data to reflect the diverse social factors in urban regions. In this process, we conducted a dimension reduction method for the public record data and used a machine learning-based clustering algorithm for the SNS data. In doing so, we found that functional distance could be defined as the Euclidean distance between social function vectors in urban regions. Finally, we examined whether the functional distance was a variable that had a significant impact on urban human mobility.

## Introduction

The latest models used to predict intra-city traffic employ physical factors, such as the population size and the distance-of-travel, as driving variables. However, because of the increasing complexity of cities, new variables have been suggested that would improve the predictive power of these models. The social function of inner regions was one such variable.

In this paper, we started by testing common models derived from the Newtonian equation for gravity but used variables other than the standard ones currently being used to resolve mass and distance. After determining the best-fitting model, we expanded it to application in functional distances, which are defined as the Euclidean distance between social function vectors of the inner regions of the city. The functional distance could be seen as a concept that indicates the social, economic, and environmental factors for human mobility in a geographic area [1, 2]. By measuring it against non-physical characteristics of the inner regions, we recommended a model in which the functional distance could be used to predict human mobility independent of physical distances.

The gravity model was useful for explaining the flow between two places. In human systems, bilateral movements in empirical studies include trade, phone calls, and transportation. Compared with the Levy-walk [3, 4], hidden Markov [5], and radiation models [6, 7], the gravity model has been shown to capture the mechanism of human movements more parsimoniously [8–14]. While there was empirical evidence supporting the use of the gravity model in predicting enduring and long-distance movements, consideration as to whether this model was effective in predicting transient and short-distance movements was warranted. Large numbers of people with a huge range of purposes for movement often come in and out of cities and move through complex transportation systems. This reality of urban life makes it difficult to predict traffic in cities solely on the basis of a traditional gravity model. To address this issue, we recommended the use of more realistic factors for mass and distance terms than the variables currently being used for long-distance movement.

Specifically, we expanded the scope of the gravity model by incorporating several socio-demographic characteristics of various places. Some of these characteristics have already been used to predict trip generation [13, 15]. In this study, we defined local functions based on public record and SNS data so as to reflect the purpose of population movement in the gravity model by employing a variable for the functional distance between regions. Based on this, we theorized that people use features in other regions that are not available in the region in which they are currently located.

In an effort to improve its predictive power, we tested the model using two large-scale sources of public and SNS data. We also compared the results using travel distance and time as alternatives to the distance term. The number of employees and the number of tweets were used as alternatives to the existing mass term of the gravity model. Furthermore, we examined the effect of functional distance on traffic volume between urban regions by allowing the extended model to take into consideration the functional characteristics of these urban regions.

Sections of this study deal with the following contents. Firstly, in the Data section, we showed the summary and features of the traffic, public records, and SNS data that were collected. Three data sources were included: the Seoul travel card data (T-Money) provided by the city of Seoul, the public record data provided by the South Korean government through a public data portal [16], and SNS data collected from Twitter. To analyze such a large volume of raw data, we used dimensionality reduction techniques that could manage the addition, modification, and removal of multiple data sources. Secondly, the Results section was divided into four parts: the gravity model in the city, the function of urban regions, functional distance, and modified gravity models using the calculated functional distance. In the first part, we compared the various mass and distance values to find out which values were the most suitable for predicting traffic volume in the city. In the second part, we differentiated the function of each urban region as either a vector of regional social factors or topics of tweets. In the third part, we defined the functional distance as the Euclidean distance between these vectors. Finally, we proposed a modified gravity model that increased the accuracy of urban human mobility prediction by searching for correlations between the functional distance and urban traffic.

## Related studies

### Human mobility in city

In this study, we used the gravity model as a representative model for trip generation and distribution, which are both stages of the four-step model for traffic prediction (described below). While the gravity model demonstrated good predictive capacity across various applications, there was a question of whether the model could clearly explain urban traffic. To resolve this question, we identified the ranges, masses, and distances of existing gravity model-based studies before selecting the most suitable range and values for this study.

In existing long-distance studies, we noted that the model was highly accurate when used to define residential population or economic index as the mass value, and the straight-line distance between the two points as the distance value. Examples of such long-distance studies include the prediction of trade volume based on income level or GDP between countries [17, 18] or the prediction of traffic flow between cities based on population [12, 14]. Studies that focused on estimating urban traffic volume used mass values such as resident population, flow population, and the number of employees [13], as well as distance values such as IVTT (In-Vehicle Travel Time), OVTT (Out-Vehicle Travel Time), and cost [19, 20]. In this current study, we present a modified gravity model that better explained urban human mobility by comparing a number of potential variables to be used as the gravity and distance terms.

Additionally, it was necessary to clarify the scope of the study through an overall understanding of the four-step model. This model was used to predict traffic during the following phases: Trip Generation, Trip Distribution, Mode Choice, and Route Assignment [21]. In the Trip Generation step, possible traffic volume was calculated by considering features such as the resident population, the number of employees, and the income level of the origins. In the Trip Distribution phase, traffic volume was allocated to each destination. In the Mode Choice step, the transportation mode to be used was determined. Finally, the Route Assignment step was conducted to determine which path to move through.

Firstly, in order to improve the accuracy of the Trip Generation stage, various factors, including social ones, had to be considered. There are existing studies that have considered social factors such as the number of employees and people in the household [22], accessibility of transportation modes [23–29], and population density [30–32]. However, the significance and weight of each variable changed depending on the prevailing circumstances in which the traffic volume was predicted. Also, these studies did not cover all the variables mentioned. Therefore, there was the need for a methodology that could integrate a comprehensive set of factors which could be used to determine trip generation without being affected by any predominating circumstances.

For the Trip Distribution phase, existing studies have improved their performance through regular updates to the model. After the 1950s, several mathematical analysis-based models were presented, including gravity [33–35], intervening opportunities [36], and hybrid models [37–39]. These studies are commonplace in that they are based on the characteristics of two regions, including the population and the distances between these two regions.

Recent studies have focused on SNS data as a proxy for human mobility. Human mobility patterns extracted from geo-tagged tweets could be used one of these proxies [40]. These patterns could also be applied to improve human mobility prediction models, such as the Gravity and Radiation models [41]. Moreover, some studies [42, 43] tried to determine the locations of SNS data without geo-tags because the number of SNS data containing geo-tags was limited. However, these studies did not focus on the context of SNS data as a predictor of human mobility. In this work, improved methods for both the trip generation and trip distribution steps were recommended. We wanted to investigate whether the context of SNS data was a valid dataset that could be used to predict trip generation, and if using the functional distance could improve the model for the trip distribution step. These approaches could be used to describe the function of urban regions and the relationships that exist among them with easily collected data. We have defined the function of urban regions in the next section.

Recent studies have focused on SNS data as a proxy of human mobility. Extracted human mobility patterns from geotaged tweets can be one of them [40]. These patterns also can improve the human mobility prediction models including the gravity model and the radiation model [41]. Moreover, some studies [42, 43] tried to find out the locations of SNS data without geotags because the number of SNS data with geotags is limited. However, these studies did not focus on the context of SNS data as a predictor of human mobility.

In this work, we suggest improved methods for the trip generation and trip distribution step. We investigated whether the volume of SNS data is a valid data set that can predict trip generation and if using the functional distance can improve the model of trip distribution step. These approaches can help describe the function of urban regions and relationships among them with easily collected data.

### The function of urban regions

Even though it has been improved through the application of refined measures for mass and distance, the gravity model is considered to be elegantly simple albeit over-generalized. Given that human movements tend to be purposeful [4, 44], by paying heed to the reasons for human purpose, we could build up a more realistic model for human mobility. The modified gravity model proposed in this study utilized a functional distance based on the functions of each region inside the city.

Functional distance is a theoretical device used for studying human mobility and interaction. With full recognition of the physical and geographic factors, it calls for attention to the effects of social factors (net physical constraints) on the interactions among various places. While properly taking into account the significance of the non-physical factors in human mobility, the concept of functional distance was not deemed as a predictor of human mobility. Rather, it was considered as an outcome of human mobility. For instance, in their study on “urban hierarchy”, Brown et al. presupposed that functional distance was not a condition, but rather an outcome of the social characteristics of the places and the migration patterns conditioned by them. As migration volume was included in it, the lower functional distance indicated a great level of interaction between the origin and the destination, while the “place utilities” of the two places were dissimilar [2]. In a similar vein, Stutz made a distinction between social and functional distance. The social distance measures the degree to which people living in separate areas differ in wealth, occupation, ethnicity, political attitudes, and housing. “As physical and social distances increase between participants, interaction is likely to decline” [45]. Functional distance appears to be a result of physical and social distance.

However, functionally distant areas are not always internally homogenous. A functional region is internally differentiated into its center and hinterlands. Human mobility within the area is most frequent between the differentiated sub-areas [46]. Therefore, when we considered the functional distance as a determinant rather than a result of human mobility, we did not need to stick with the classic definition of functional distance as this contained the volume of human mobility and also appeared to be an outcome of it. In so doing, we were able to better understand the flow of people across urban areas of “organizational, economic, and cultural differences” [47]. Furthermore, it enhanced our understanding of how social and technical factors influenced human mobility and the structure of urban spaces [48].

Commuting was one primary example of our problem [49, 50]. In order to take the purpose of individuals’ transit into account in the gravity model, existing studies used the mass value as the possible traffic generated in a particular region, and calculated it by using several variables (including social factors). However, they did not take into consideration the functional difference between the two regions, which could be used to help infer the purpose of movement. People move because they need a function from the destination which they cannot obtain at the origin.

Various studies have defined the functions of urban regions. Zoning is one of representative methodologies for doing this. The United States began broadly using zoning for city planning after the Euclid judgment (Village of Euclid v. Ambler Realty Co., 272 U.S. 365, 1926). This approach gave legal status to urban regions designated as commercial, residential, industrial, and special zones, each with its own sub-categories. Unfortunately, it was difficult to explain old and complex cities with Euclid zoning because this concept arose from modern city planning. In the case of Seoul, for instance, with its 600-year history, 89% of commercial areas have been designated as “General Commercial Areas” and 43% of residential areas have been designated as “Second Type General Residential Areas,” thus resulting in a data-loss for the defining characteristics of that region. To compound the problem, the recent rise in large multi-purpose buildings introduced new difficulties to the otherwise black-and-white Euclid zoning model.

To address these limitations, a method was needed to incorporate more complex functions of contemporary urban regions. Zoning in New York City, for example, has been modified to include more granular and complex functions [51]. When we looked at functional zones in New York City in detail, there were 44 types of residential areas based on population density. The commercial function now has eight middle categories and 73 detailed categories. New York’s classification system provides a more complex and flexible way to reflect the reality that one building or region often has multiple functions. This model successfully demonstrates the increasing complexity of urban areas. A smart-zoning policy based on “White Paper on Smart Growth Policy in California” [52] and passed in California was another example of zoning that reflected rising urban complexity. This policy focused on high-density and mixed-use developments, and sought to alleviate the problems posed by traditional zoning practices. It defined the function of urban regions with more contextual factors, such as neighborhood/commercial, main street residential/commercial, urban street residential/commercial, office convenience, office/residential, shopping mall conversion, retail region retrofit, live/work, studio/light industrial, hotel/residence developments, and parking structures with ground-floor retail. These examples indicated that it was difficult to exclusively label one region with only one function. Due to changes in the distribution of functions throughout the regions, a change in zoning methods was inevitable.

Traffic in a city with traditional zoning is generally defined as movement between residential and commercial or industrial regions, whereas traffic in cities with more complex zoning occurs between mixed-use areas. Based on the increasing complexity of cities, it was apparent that we needed a modified definition of functions that could describe the roles of regions with updated nuances rather than simply relying on the traditional zoning scheme.

In defining functions within a city, not only should the physical factors be reflected, but also the social factors. Social factors have not been exhibited by the existing zoning methods, even though the behavior of residents is often affected by race [53], income [54], and education [55]. For example, people’s tendencies to be self-isolated in their homes is often influenced by the race of their neighbors [53]. For the particular purpose of this study, it was important to note that social-demographic features, including marital status, education level, and age, influence commute times [56].

Recently, it is possible to examine functions of urban regions not only with traditional social data, but also with data that can be collected in large quantities. Toole et al. predicted zoning regulation of lands with mobile phone activity records [57], Qi et al. showed the social functions of each region by using taxi behavior [58], and Yuan et al. discover different functions of regions based on POI data [59]. These studies analyzed the functions of urban regions with a huge data set, but they did not suggest practical uses for their results.

Moreover, existing studies that have tried to understand the functions of urban regions using geo-tagged SNS data, have revealed complex factors in urban regions. The study from Giridhar et al. detected events in the city with Instagram data [60]. Jiao et al. showed that the number of tweets with spatial reference was only a small proportion of the total number of tweets generated, and also determined the relationship between spatial tweets and a special event [61]. Hasan et al. predicted urban activity patterns via online geo-location data [62]. They classified the topics of tweets as activity categories containing similar topics in order to show temporal activity patterns. However, detailed analyses of the regions and the complex relationships among them were not determined. Nevertheless, these studies proved that SNS data could be used to reveal both complex and detailed features of specific regions, including characteristics, detailed emotions, and the activities of people who visit or live there; things that the public record data could not explain on its own. Taking these studies into consideration, we proposed a modified gravity model as the best way to predict intra-city traffic. The goal was to improve upon existing urban traffic prediction models, which only included limited data sets and gave consideration for terminology details. More detailed descriptions have been provided in the following sections.

## Data

### Human mobility data (T-Money)

In this study, we used smart card data obtained from Seoul’s public transportation system to track human mobility. Studies in human mobility have incorporated various types of data, such as cargo-ship movements [63], automobile traffic flow on highways [12], and mobile phone records [64–66]. Smart card data offered detailed and accurate records on urban human mobility. The data took into account the structure of both bus and subway lines as well as other factors, including the occurrence of traffic jams which ultimately characterized public transportation use.

The public transportation system in Seoul is one of the most complex systems in the world. As of 2017, it consists of 20 subway lines and 360 bus lines. There are about 10 million daily movements by 5 million passengers. To account for the high volume, T-Money data is used. T-money is the brand name of Seoul’s travel card. T-money cards cover more than 95% of public transportation use in Seoul. These cards record travel as a trip-chain that includes information about transits as well as the departure and destination places. These are then used to set prices based on information about distance and transfer. Hence, every point in time and place regarding the traveler’s movements can be tracked.

Unlike GPS-based location data which track the trajectory of travelers, smart-card data contain less detailed information because they record only station locations with time stamps of when a passenger embarks or disembarks [5]. However, smart-card data are more efficient at compiling movement records because they do not require additional sensors. Common peaks in traffic volume within the distance (3 km) and time (10 minutes) have implied that traffic, distance, and time were strongly associated.

**Fig 1:**
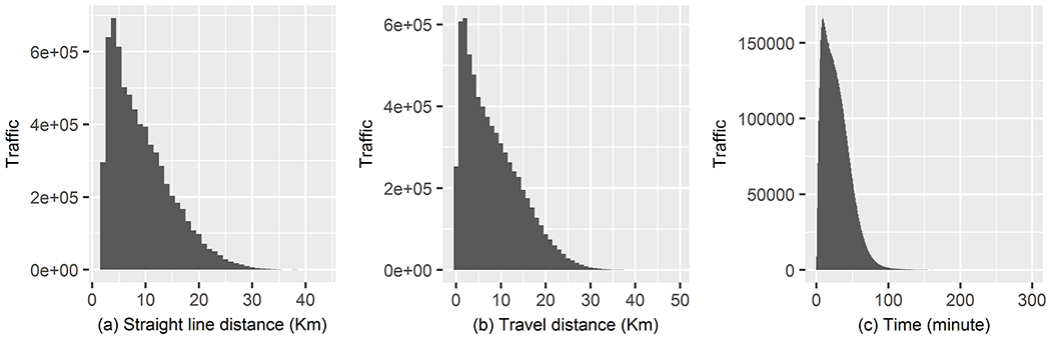
Distribution of distance values. (a) Straight-line distance, (b) Travel distance, and (c) Time spent in Seoul public transportation system.

There were several tasks that needed to be completed before the public record and T-Money data could be applied to the urban human mobility study. First, missing cases in the distance data have to be filled in. There were three types of distance terms in this study, including the straight-line distance, the average travel distance, and the average time between two regions. It was possible to make a complete dataset with the straight-line distance because the center positions of all regions were known. However, in cases of travel distance and time, there were many missing cases. It was possible to fill in the travel distance and time cases from the straight-line distance because they had strong positive relationships with Pearson coefficients of more than 0.6. More specifically, Fig 1 showed their distributions in Seoul, and Table 1 showed linear regression results used to predict the missing travel distance and time data from the straight-line distance. This was done by using average values obtained between basic administrative districts. While it was not possible to calculate the exact average travel distance and time among regions, the statistically significant results of both models indicated that they could be used to fill in the missing cases.

**Table 1:** Linear regression results of distance terms.

Linear regression results		
	Dependent variable:	
	(1) Travel distance	(2) Time
Straight-line distance	1.089***	0.161***
Constant	2,297.914***	1,165.428***
Observations	81,543	81,543
*R*^2^	0.785	0.610

*p < 0.1

**p < 0.05

***p<0.01

Additionally, we converted longitude and latitude coordinates in the datasets into addresses because the public records did not contain detailed coordinates, unlike smart card data and SNS data that contained exact coordinates. Public records were divided into basic administrative districts called a “Dong”, so as to convert coordinates into Dong via a SHP file. Therefore, analysis in this study was conducted via Dong.

### Public record data (Government 3.0)

To define the functions of urban regions, we used the public records data provided by the government of South Korea. The data reflected diverse characteristics in urban areas and could be effectively used to calculate functional distances. These kinds of public record data have been increasing in quantity and type, and various city plans have been made based on them. As of 2016, the government of South Korea has provided approximately 4,000 types of data through public APIs and file downloads from the public data portal website [16]. Data on Seoul have been provided up to the basic administrative district level. There are 423 basic administrative districts in Seoul, with an average of 23,000 inhabitants in each area. Overall, the city has a population of about 10 million, including 3 million employees and 3.5 million households. Table 2 shows detailed information on the public records used in this study.

**Table 2:** The number of datasets by type.

Type	Detail	Count	Total
Households	The number of households by the number of members	8	50
	The number of household members	20	
	The number of households	7	
	The number and rate of female householder households	3	
	Diffusion ratio of house	3	
	The number of houses by type of housing	9	
Population	Elderly person statistics	12	38
	Move in /out	8	
	The number of residents (gender/age/foreigner)	12	
	Birth / death	6	
Business	Business status	130	199
	Year of establishment of business	24	
	Status of women’s businesses	31	
	Density of business and workers	7	
	Start-up ratio	4	
	Average age of workers	3	
Welfare	Elderly welfare facility	4	100
	Basic livelihood security recipients	6	
	The elderly living alone	24	
	Childcare facilities / workers	27	
	Medical facilities / workers	39	
Tax	Tax (property/income/consumption/acquisition tax)	15	15
Administration	Administrative district (number, area)	6	15
	Civil servants (classification and number)	9	
	Total sum	417	

As mentioned before, a basic administrative region of South Korea is referred to as a “Dong.” Since each Dong serves as the official boundary for community centers and electoral regions, the administrative bodies of the various cities set up the populations of these units as evenly as possible. However, for practical reasons (including the area’s geography), the population distribution in particular administrative regions follows a gentle, normal distribution curve instead of the usual uniform distribution. Larger infrastructure systems, such as university hospitals and train stations, do not need to exist in every region. As such, this causes functional differences among the urban regions. To reflect these situations, a modified gravity model was set up based on functional distances, including functional differences and biases.

### SNS data (Twitter)

SNS data was another dataset used to calculate the functional distance as it reflects people’s lives in particular locations. Twitter is one of the most popular microblog SNS because it not only has text, image, and movie contents, but also geo-location data. Even though all tweets do not contain geo-location data, many Twitter users upload the geo-location data automatically generated by their mobile devices.

An understanding of the characteristics of Twitter users was required to determine the functions of urban regions in tweets. It was possible to catch users’ location, gender, race, and language from the metadata of tweets [67–69], and infer the users’ occupation based on text analysis [70]. As a result, about 40% of Twitter users tended to have lower managerial, administrative, and professional occupations, and their age ranged from the late preteens to the early 30s. When we consider more details about the users who engage geo-location services, 41.6% of users enabled a location service, and 3.1% of users turned on the geo-tagging service [71]. However, it was difficult to argue that there were significant difference between users who used geo-location services or not. Therefore, we assumed that the users’ characteristics in our dataset were the same as those of typical Twitter users.

First, we collected all the IDs of tweets generated near Seoul in 2013 through a python-based tweet crawler. We obtained detailed information using Twitter API and collected IDs. This was done to understand the functions of various urban regions from April 12 to April 14, 2013, the period during which public transportation data was collected. As a result, we used only tweets that contained accurate location information near Seoul, thus 654,776 tweets that had unique IDs, user IDs, texts, creation times, and geo-location data were collected. After that, we converted the tweets’ coordinates to basic administrative districts. This was an essential process in unifying the unit of analysis.

## The gravity model in the city

### Gravity equations

The gravity model, as derived from Newton’s law of gravity, has served as an excellent predictor of social phenomena such as immigration, communication, and trade [17, 18, 72]. According to Eq (1), traffic between two regions is proportional to the product of the mass values in each region and inversely proportional to the distance between the two regions. The main components of the gravity model for human mobility studies are Traffic (*T*), Mass (*M*), and Distance (*D*). *T*_*ij*_ is the volume of mobility between areas *i* and *j*, whereas *M*_*i*_ represents the mass of area *i*. In human mobility prediction models, *M* is the population of areas, whereas *D*_*ij*_ represents the geographical distance between areas *i* and *j*. Existing studies have shown that gravity models could be used to explain long-distance cases quite well, with positive *α*_1_ and *α*_2_ values and a negative *α*_3_ value [8].

$$\begin{array}{c} T_{ij}=\alpha_{0}M_{i}^{\alpha_{1}}M_{j}^{\alpha_{2}}D_{ij}^{\alpha_{3}} \end{array}$$ (1)

Since urban mobility was different from long-distance mobility, it was necessary to find suitable values for the gravity model terms in the city. The resident population [7, 72] and the number of employees [8] were values for mass terms used in existing studies. Residential population was a strong predictor of long-distance human activity, but because it was not guaranteed to be accurate in cities, it was necessary to compare various mass values in order to determine the most suitable one. For the distance term, straight-line distance [17, 18, 72], time, and travel distance [73] were possible values.

Eq (2) is one of the multiplicative gravity models used in this study. Eq (3) is a log-linear form of Eq (2). Multiple mass values, including residential population (*RP*), the number of employees (*EMP*), and the number of tweets (*TW*), as well as multiple distance values, including the physical distance (*D*) and the functional distance (*FD*) were used in these equations. A detailed definition of the functional distance has been covered in the functional distance section.

$$\begin{array}{c} T_{ij}=\beta_{0}{\text{RP}}_{i}^{\beta_{1}}{\text{RP}}_{j}^{\beta_{2}}{\text{EMP}}_{i}^{\beta_{3}}{\text{EMP}}_{j}^{\beta_{4}}{\text{TW}}_{i}^{\beta_{5}}{\text{TW}}_{j}^{\beta_{6}}D_{ij}^{\beta_{7}}{\text{FD}}_{ij}^{\beta_{8}}\varepsilon_{ij} \end{array}$$ (2)

$$\begin{array}{ccc} lnT_{ij} & = & \beta_{0}+\beta_{1}ln{\text{RP}}_{i}+\beta_{2}ln{\text{RP}}_{j}+\beta_{3}ln{\text{EMP}}_{i}+\beta_{4}ln{\text{EMP}}_{j}\\ & & +\beta_{5}ln{\text{TW}}_{i}+\beta_{6}ln{\text{TW}}_{j}+\beta_{7}lnD_{ij}+\beta_{8}ln{\text{FD}}_{ij}+\varepsilon_{ij} \end{array}$$ (3)

However, the traditional gravity equation does not usually consider multilateral resistance terms. Anderson and van Wincoop suggested that exporter and importer fixed effects could solve this problem [74]. Moreover, constant-elasticity models with a non-linear estimator were recommended for solving zero-value observations.

According to Anderson and van Wincoop, it was possible to state that the traffic between region and had a Poisson distribution with a conditional mean (*μ*) that was a function of independent variables (Eq (4)). Here, conditional mean *μ*_*ij*_ is described by an exponential function of the regression variable, *X*_*ij*_ (Eq (5)). Additionally, *α*_0_ is a proportionality constant, and *β* is a corresponding parameter vector. *ε*_*i*_ and *γ*_*j*_ are effects specific to the origin and destination region, respectively.

$$\begin{array}{c} Pr\left[T_{ij}\right]=\frac{\exp\left(-\mu_{ij}\right)\mu_{ij}^{T_{ij}}}{a},\left(T_{ij}=0,1\ldots \right) \end{array}$$ (4)

$$\begin{array}{c} \mu_{ij}=\exp\left(\alpha_{0}+\beta^{{}^{\prime }}X_{ij}+\varepsilon_{i}+\gamma_{j}\right) \end{array}$$ (5)

Santos Silva and Tenreyro suggested Poisson Pseudo Maximum Likelihood (PPML) estimator as a non-linear estimator to solve the inconsistency problem caused by heteroskedasticity of the Ordinary Least Square (OLS) estimator. Burger et al. [75] challenged the idea that PPML was vulnerable to the problem of overdispersion in the dependent variable and excessive zeros [76]. They suggested using Negative Binomial Pseudo Maximum Likelihood (NBPML) instead of PPML to solve the overdispersion problem in the dependent variable. They also suggested using the Zero-Inflated Pseudo Maximum Likelihood technique (ZIPML) and the Zero-Inflated Binomial Pseudo Maximum Likelihood technique(ZINBPML) because these techniques held up in the presence of excessive zeros.

The Zero-Inflated Model considers the existence of two latent groups within the population: a group that had strictly zero counts and a group that had a non-zero probability of having counts other than zero [75]. In the case of the Zero-Inflated Negative Binomial Regression Model defined in Eqs (6) and (7), *ψ*_*ij*_ is the proportion of observations with a strictly zero count (0 ≤ *ψ*_*ij*_ ≤ 1) which was determined by a logit model.

$$\begin{array}{c} Pr\left[T_{ij}=0\right]=\psi_{ij}+\left(1-\psi_{ij}\right){\left(\frac{\alpha^{-1}}{\alpha^{-1}+\mu_{ij}}\right)}^{\alpha^{-1}} \end{array}$$ (6)

$$\begin{array}{c} Pr\left[T_{ij}\right]=\left(1-\psi_{ij}\right)\frac{\Gamma (T_{ij}+\alpha^{-1})}{T_{ij}!\Gamma \left(\alpha^{-1}\right)}{\left(\frac{\alpha^{-1}}{\alpha^{-1}+\mu_{ij}}\right)}^{\alpha^{-1}}{\left(\frac{\mu_{ij}}{\alpha^{-1}+\mu_{ij}}\right)}^{T_{ij}} \end{array}$$ (7)

Spatial autocorrelation is another important problem of the gravity model. Characteristics of urban regions and interactions among them tended to affect those of nearby regions. To solve the spatial autocorrelation problem caused by the proximity of the physical distance, some other types of models have been proposed. The Spatial Autoregressive model (SAR) captures spatial dependence by using the weight matrix. The Eigenfunction Spatial Filtering (ESF) approach represented an alternative methodology to account for spatial autocorrelation in a spatial interaction model. This ESF approach was recommended for points data by Griffith [77], and it was also extended for application to links data [78–80].

SF decomposition is a transformation procedure based on eigenvector extraction from Eq (8), where *W* is a generic *n* × *n* spatial weight matrix, *I* is an *n* × *n* identity matrix, and 1 is an *n* × 1 vector containing 1s. Extracted eigenvectors were selected by using Moran’s coefficients(MC) with the following threshold: *MC*(*ϵ*_*i*_)/*MC*(*ϵ*_1_) > 0.25. We called these selected eigenvectors “candidate eigenvectors”. After that, we only utilized statistically significant eigenvectors among the candidate eigenvectors by using a stepwise regression model. This was called a “Spatial Filter”. To apply the spatial filter into the flow between points like traffic, it was necessary to use Kronecker products. *E*_*K*_ ⨂ 1, where *E*_*K*_ is the *n* × *k* matrix of the selected eigenvector, is linked to origin specific data, and 1 ⨂ *E*_*K*_ is linked to destination specific data.

$$\begin{array}{c} \left(I-11^{T}/n\right)W\left(I-11^{T}/n\right) \end{array}$$ (8)

Considering the distribution of dependent variables in our data, we used the ZINBPML estimator when considering the distribution of data. Moreover, we needed to use ESF to remove spatial autocorrelation, so that all models in this work followed the ZINBPML ESF model.

### Mass

The residential population is the most commonly used mass value in gravity models of traffic flow for human mobility studies [12, 72]. However, to ensure that this value worked well for shorter mobility such as those within a city, we reviewed it in alongside two other values for this study, and included the number of workers and the number of tweets. The fact that a city generally has a large residential population implies that the city will also have sizable economic, industrial, and cultural infrastructure. Therefore, in this case, although resident population could function as a close indicator of the traffic volume among cities, it was difficult to say whether it played the same role in population migration within the city.

**Fig 2:**
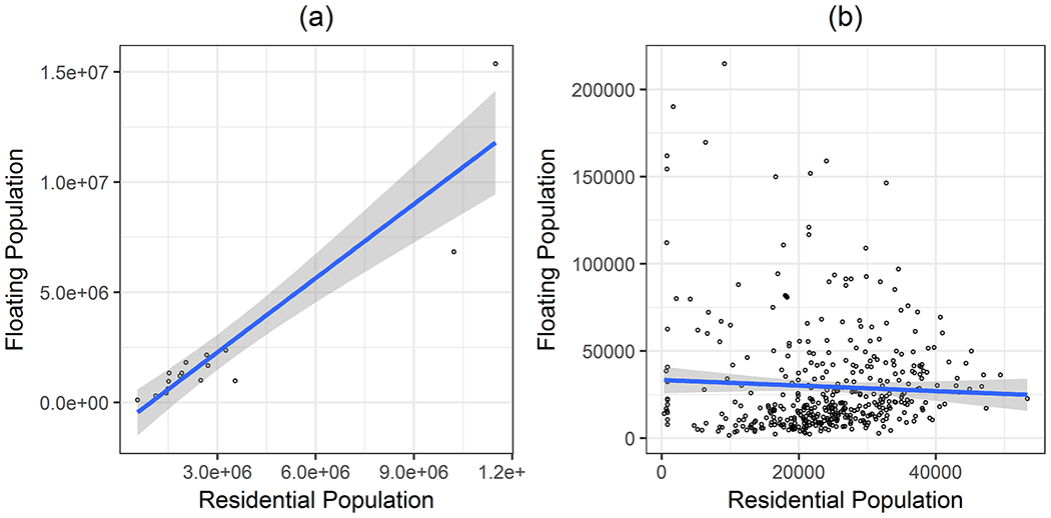
The relationship between residential population and floating population. (a) Inter-city cases, (b) Intra-city cases.

We compared the relationship between the resident population and the floating population in order to find differences between inter-city and inner-city cases. The floating population of an area is defined as the sum of the traffic originating from the area and the traffic arriving into the area. In Fig 2a, each case indicated the presence of wide administrative districts. There was a strong proportional relationship between the two population values, but this was not true in Fig 2b which plots inner-city regions. In the case of inter-city human mobility, the correlation between the residential population and the floating population was clear, but not in the inner-city case. Since each city incorporated various functions such as economy, industry, and culture, it was not easy to define cities using a single function. However, urban regions often had clear functional characteristics, such as residential regions, industrial regions, and business regions. In other words, the fact that the city had a large residential population meant that there were many other infrastructures that also caused a large amount of traffic. On the other hand, it was difficult to predict the traffic volume simply via the residential population because some urban regions had an important role, even if there was not a sizable population of residents.

The top-left cases in Fig 2b show the business regions with a small residential population, but a large floating population. It was important to note that these results clearly showed that key functions of non-resident regions had a significant impact on the floating population, and that these cases were not often present in the wider administrative districts. This is natural, considering the physical constraints of distance and time, because the city has to be formed within a reasonable range of time from residential and other regions. Residential population closely related to home-based trips can also affect urban human mobility. Therefore, it was necessary to compare several possible values, including the residential population, to find the optimal result. Goh et al. suggested that the number of employees and the floating population could be used as new mass values for the city [8].

The number of employees was another important factor that could cause urban traffic. This number was closely related to the commuting patterns of mainstream human movements on a weekday morning from residential regions to business, commercial, and industrial regions. Moreover, it was possible to use the floating population as a mass value in the gravity model because it was an obvious value that showed the activation degree of regions in the city. Even though the floating population was a powerful value that could be utilized to predict the traffic between regions, a tautology problem was observed when we used the floating population as a mass term of the gravity model, considering that the definition of floating population contains all traffic related to that area. For this reason, we compared three mass values, including the residential population, the number of employees, and the number of tweets.

Table 3 show the results of gravity models with various mass values and the public transportation traffic data for Seoul. The residential population, the number of employees, and the number of tweets were used as mass terms of the models in Table 3 (1)-(3).

**Table 3:** Regression results of different mass value models with ZINBPML estimator.

	*Dependent variable:*			
	Traffic			
	(1)	(2)	(3)	(4)
Origin residential population (log)	0.829***			−0.103***
	(0.015)			(0.013)
Destination residential population (log)	−0.015*			−0.055***
	(0.008)			(0.008)
Origin employee (log)		0.820***		0.343***
		(0.007)		(0.007)
Destination employee (log)		0.711***		0.237***
		(0.004)		(0.005)
Origin tweet (log)			1.133***	0.878***
			(0.006)	(0.008)
Destination tweet (log)			0.931***	0.768***
			(0.004)	(0.006)
Straight-line distance (log)	−1.277***	−1.303***	−1.298***	−1.297***
	(0.007)	(0.006)	(0.006)	(0.006)
Constant	8.494***	3.122***	0.309***	0.052
	(0.175)	(0.091)	(0.078)	(0.153)
Observations	178,929	178,929	178,929	178,929
Log Likelihood	−959,074.100	−938,456.900	−924,140.200	−924,871.700

*p < 0.1

**p < 0.05

***p < 0.01

By comparing log likelihood values, a model with the number of tweets was found to be the most appropriate value among the candidates. The number of tweets is closely related to the number of people in a given region, thus making it a powerful value for human mobility prediction in the city. In the case of our study, this result differed from those of existing studies, in that the explanatory power of the residential population was the most important variable in long-term cases, and long-distance mobility was lower than in other cases. Otherwise, the explanatory power of the residential population was lower than other values. However, considering that all mass values were statistically significant, it was reasonable to use the model with all mass values even if their explanatory power differed. A model with three mass values (Table 3 (4)) showed that all mass values were still significant, even if they were in a model.

There was an interesting observation regarding the signs of coefficients. The number of employees and the number of tweets always had positive coefficients, and the straight-line distance had negative coefficients. This was similar to the results obtained from the traditional gravity model. However, the residential population had either negative or no significant coefficient in several models. In Fig 2b, we have already shown that there were many regions with a large floating population and those with a small residential population. If there was a lot of traffic between those regions, it would be possible to explain the coefficients noted for the residential population. In the following sections, we have modified and expanded Table 3 (4) to improve the traffic gravity model in the city.

### Distance

Distance was another term used in the gravity model for the city’s traffic. Previous reports [12, 14, 17], as well as the results obtained from the previous sections of this study, showed that gravity models using straight-line distance worked well for inter-city traffic. However, it was difficult to argue that coordinate-based, straight-line distance presented the best representation of urban spatial structures when both the terrain and transportation systems were considered. This was because coordinated-based, straight-line distance did not take into account additional factors that arise from intra-city traffic, such as the system’s structure and traffic congestion. To solve this problem, this section compares the straight-line distance, travel distance, and time to find better variables for the gravity model of traffic in the city.

**Fig 3:**
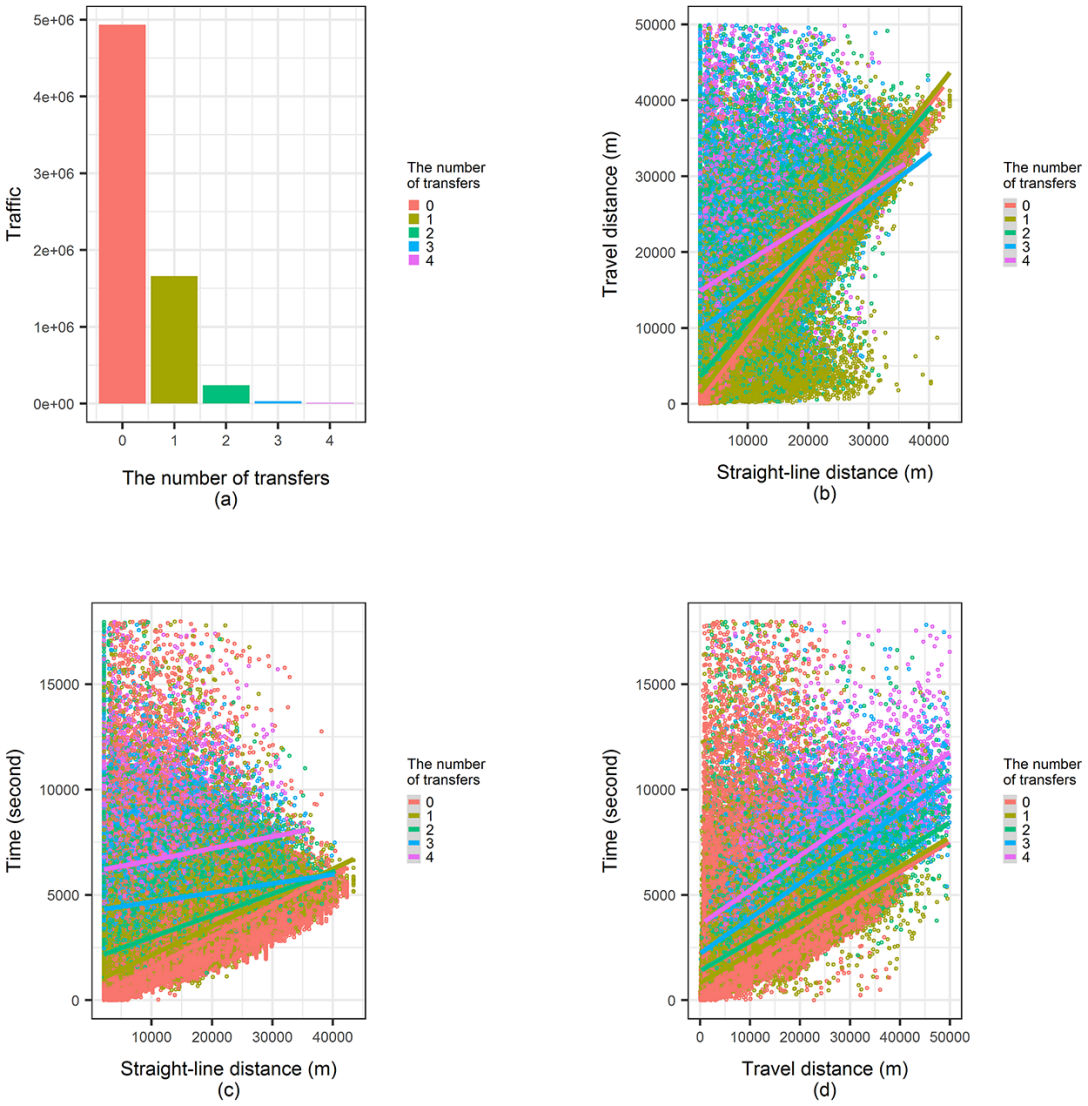
Transfer number distribution and correlations between transfer numbers and distance values. (a) Transfer number distribution, (b) Straight-line distance-travel distance plot according to transfer numbers, (c) Straight-line distance-time plot according to transfer numbers, (d) Travel distance-time plot according to transfer numbers.

Before applying the various distance variables to the gravity model, we needed to look at the relationship between these variables. As mentioned in Table 1, there were strong positive relationships between the variables, but it should also be noted that there were some differences present due to the structure of the transportation system. The transfer between public transit vehicles was seen as a typical root cause for this difference. In Fig 3, we can see transfer distributions and the difference in each distance variable based on the transfer numbers in the Seoul public transportation system. Fig 3a shows the transfer distribution, which only includes bus-bus and bus-subway transfers, and does not factor in transfers between subway lines. This shows that about 30% of passengers carried out a transfer and the rate tended to increase even further if they included a subway-subway transfer. The travel distance reflected the structural features of the system, including transfer. Time could be used as a variable to reflect the waiting time and traffic congestion that occurred during transfers.

Fig 3b-3d are scatter plots that show the relationship between straight-line distance, travel distance, and time based on the number of transfers extracted from 6 million records of the Seoul public transportation system. According to these results, there was no major difference between the straight-line distance and the travel distance when the number of transfers was two or fewer or the distance was at least 30km. However, if the distance was shorter than 30 km and the number of transfers was three or more, there was a significant difference between the straight-line distance and mileage. These results showed that the number of transfers had a significant impact on travel distance, and that the metropolitan transport prediction model with complex transportation systems should be designed to reflect this. On the other hand, Fig 3d shows that increasing the number of transfers significantly increased the amount of time it took to travel the same distance. This led to the conclusion that the distance traveled may not be the best variable to show urban human mobility. Even if travel distance could be used to reflect the shape of the transportation system, there were additional factors that distance alone could not define. Based on this difference, three distance variable candidates were applied to the model and compared.

In Table 4, there are results with different distance types: straight-line distance, travel distance, and time. The log likelihood values of the models showed that time was the best independent variable as it had more explanatory power. Time reflected real-time situations, including transfer time and traffic jams, whereas travel distance only reflected the shape of the route. To define the best prediction model for our system, we used time as a distance term in the models shown in subsequent sections.

**Table 4:** Regression results of different distance value models with ZINBPML estimator.

	*Dependent variable:*		
	Traffic		
	(1)	(2)	(3)
Origin residential population (log)	−0.103***	−0.140***	−0.101***
	(0.013)	(0.012)	(0.011)
Destination residential population (log)	−0.055***	−0.081***	−0.008
	(0.008)	(0.008)	(0.007)
Origin employee (log)	0.343***	0.324***	0.296***
	(0.007)	(0.007)	(0.007)
Destination employee (log)	0.237***	0.237***	0.235***
	(0.005)	(0.005)	(0.005)
Origin tweet (log)	0.878***	0.891***	0.820***
	(0.008)	(0.007)	(0.007)
Destination tweet (log)	0.768***	0.778***	0.708***
	(0.006)	(0.006)	(0.005)
Straight-line distance (log)	−1.297***		
	(0.006)		
Travel distance (log)		−1.436***	
		(0.006)	
Time (log)			−2.201***
			(0.008)
Constant	0.052	2.274***	6.186***
	(0.153)	(0.152)	(0.144)
Observations	178,929	178,929	178,929
Log Likelihood	−924,871.700	−920,497.600	−906,080.200

*p < 0.1

**p < 0.05

***p < 0.01

## The function of urban regions

Existing studies have defined the function of regions by using land use [51, 52] or social demographics [53–55]. However, the land use classification method is often limited by the increase in urban complexity, whereas social demographic studies have only focused on the details of individual properties. For our purpose, it was necessary to systematically classify the function of urban regions while showing the functional complexity of a specific area. Fortunately, due to quantitative and qualitative increases in urban data, the possibility of precisely defining the functions of urban regions has risen. Based on this, we aimed to present integrated functions that reflected more detailed characteristics of urban regions. In this section, we have expounded on defining the function of urban regions with various variables.

**Fig 4:**
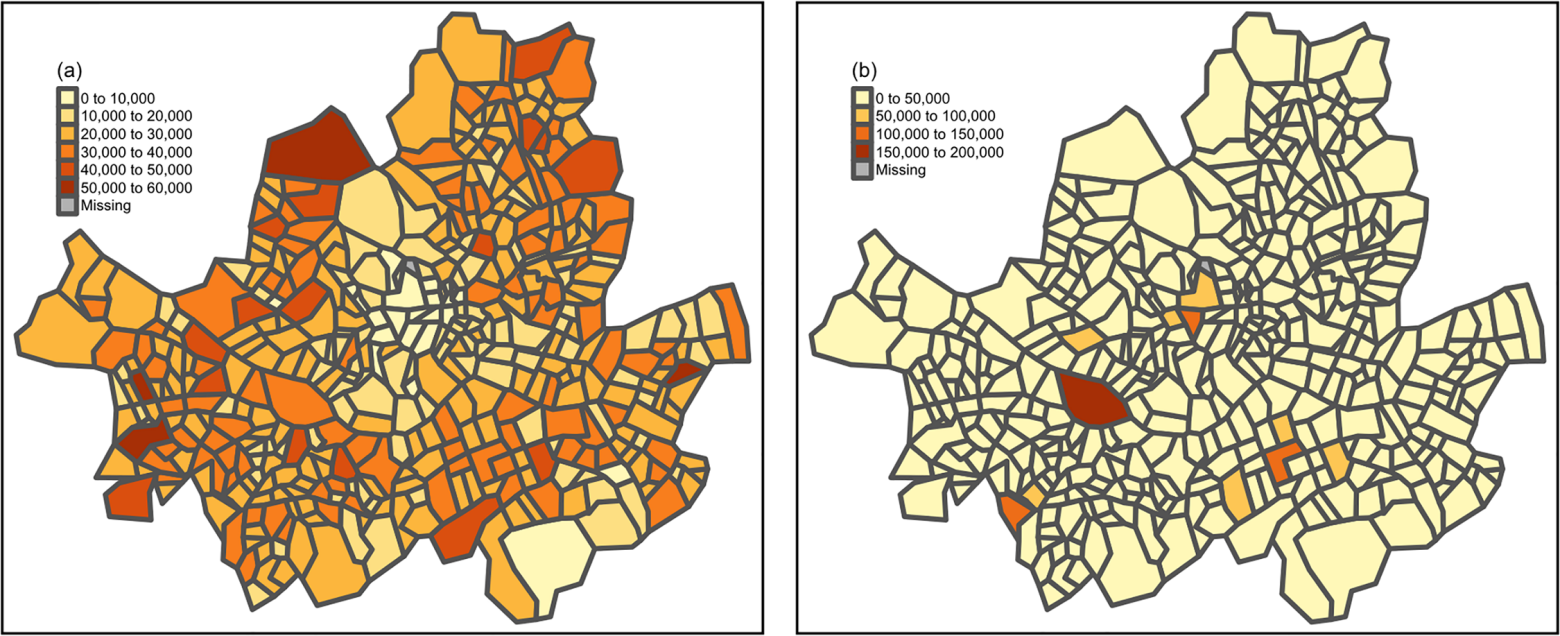
Distributions of features in Seoul. (a) Residential population (b) The number of employees.

**Fig 5:**
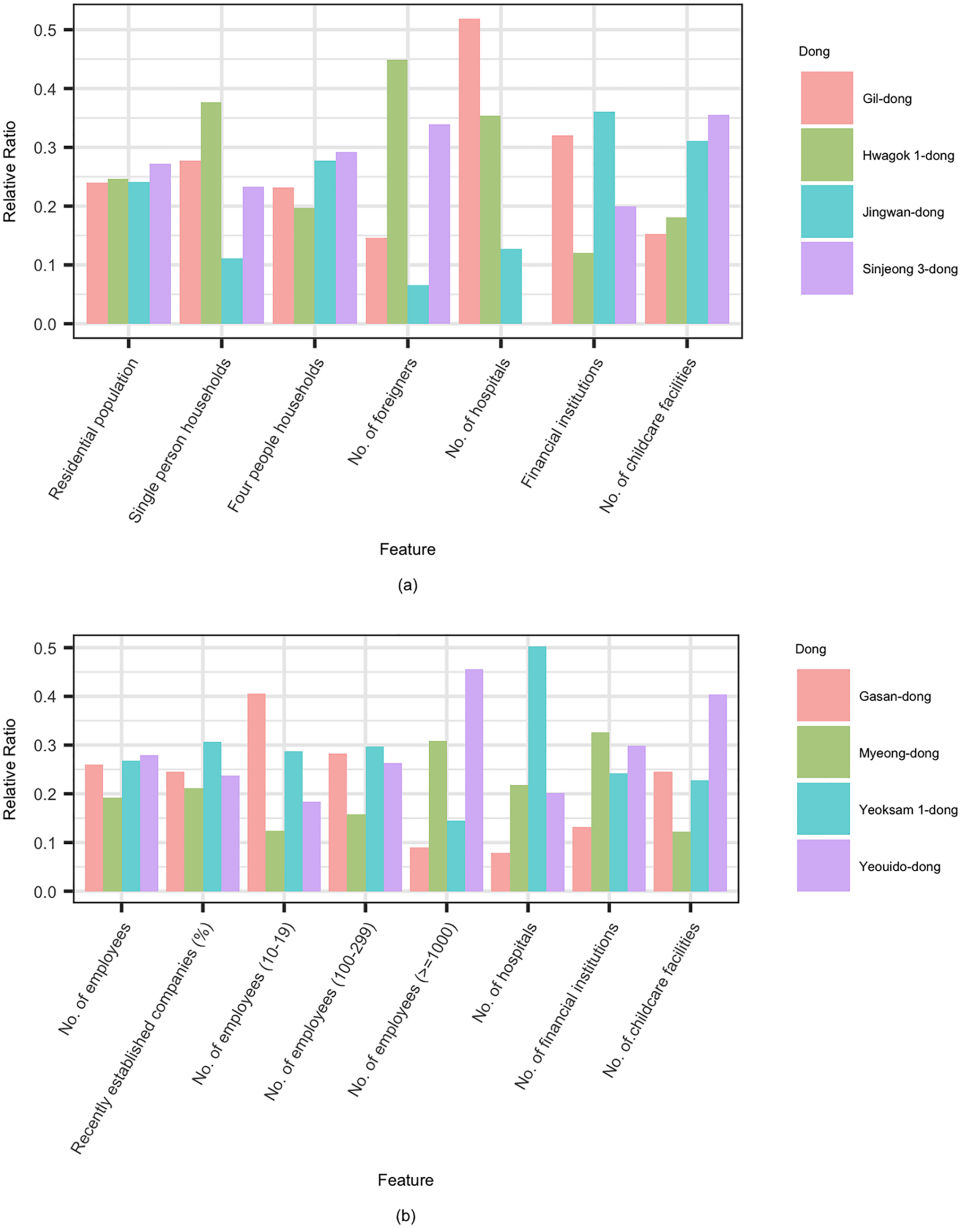
Functional differences among urban regions obtained through the public record data. (a) Major residential districts (b) Major business districts.

Fig 4 shows the distribution of residential population and the number of employees in Seoul. While the number of employees was concentrated in several business regions, the residential population was somewhat evenly distributed. We have analyzed some of these areas in more detail and showed that functions of urban regions needed to be reflected when dealing with urban human mobility problems. Fig 5a shows detailed functional differences of the main residential areas in Seoul. Although each residential area had a similar residential population, not only was there a ratio of four for single-person households to those populations, but the percentage of foreigners was also different. Moreover, there were also regional differences in the number of infrastructures such as healthcare, finance, and childcare.

Similarly, Fig 5b showed that the functional characteristics of major business districts in Seoul were also different. For example, in the case of Gasan-dong, where many IT and venture companies were located, the ratio of small-sized companies was high. On the other hand, in the case of Yeoido, where many large financial companies were located, the ratio of business companies with more than 1,000 employees was high. In addition, the distribution of medical and financial businesses were also different, depending on the business districts in which they were located.

**Fig 6:**
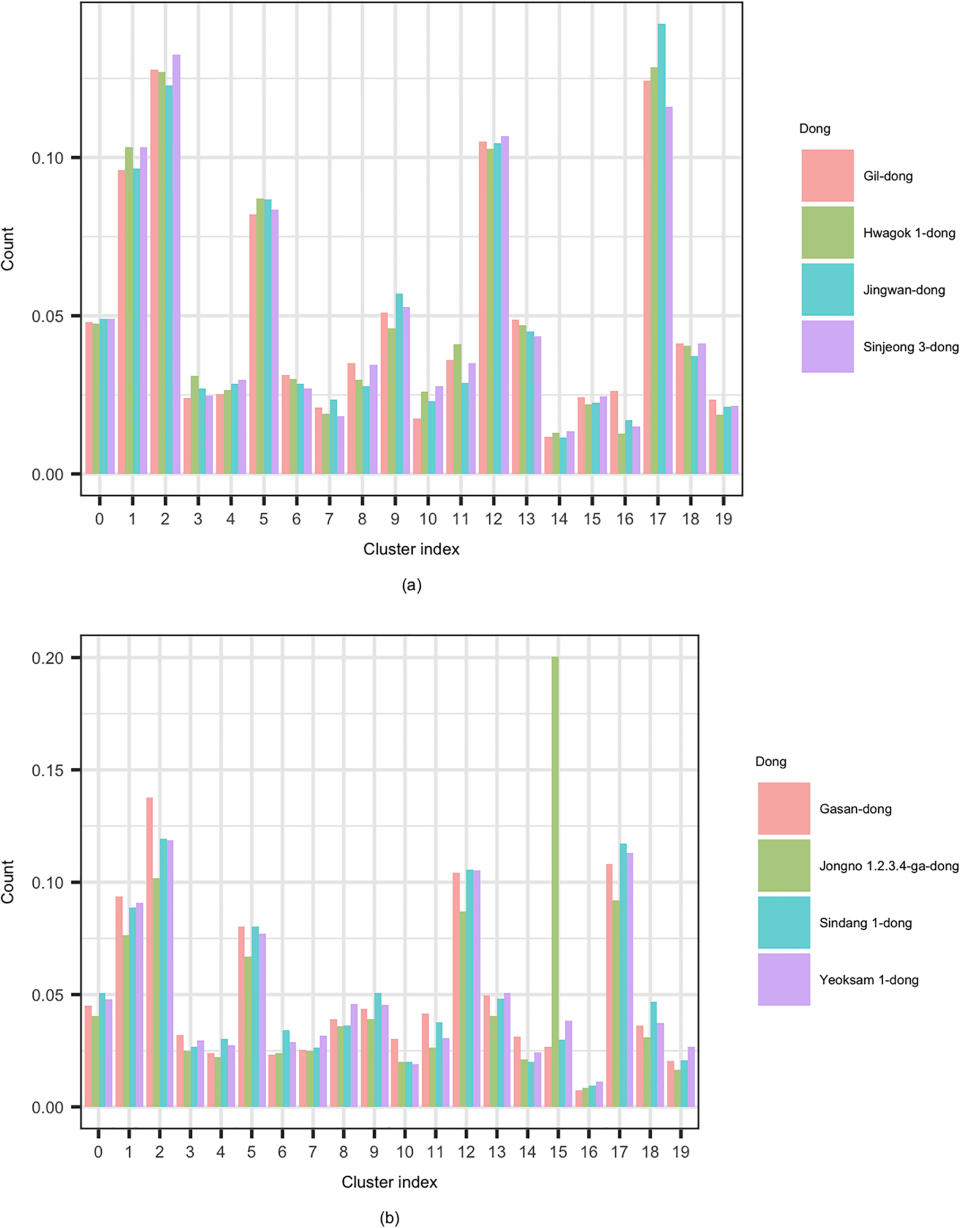
Functional differences among urban regions through SNS data. (a) Major residential districts (b) Major business districts.

Functional differences could be seen in both the public records and in the SNS data analysis. Fig 6 are graphs showing the percentage of mentions of each topic after dividing SNS data into 20 subjects. For example, even though Jongno-1.2.3.4ga dong is a business district, it also has a palace, which is a typical sightseeing spot in Seoul. Therefore, the ratio of mentioning cluster 15, including the names of major tourist attractions, was remarkably high.

Considering that urban regions conducted different functions with many points of view, we theorized that the function of urban regions needed to contain all possible features of urban regions. However, since the city was not a simple system that could be explained solely by the properties of the region itself, it has been suggested that the functional distance could reflect the functional relationships among various urban regions.

## Functional distance

As mentioned before, the function of urban regions was difficult to express with some variables. To verify the hypothesis that the functions of urban regions affected human mobility in the city, it was necessary to include numerous values that represented the functions of urban regions in our human mobility prediction model. However, the number of public records in this study was 790, and when variables from SNS data analysis were included, it was 810. Also, if this data would have been handled like the Mass term of a gravity model, the number of variables to considered would have doubled. Moreover, considering the continuous increase in the number of data types that could be used to reflect characteristics of the regions, simply adding all variables into the model did not seem appropriate. Of course, it was not entirely impossible to predict human mobility through models with a large number of variables, but we wanted to find a better solution.

For this, we focused on traffic volume as an inter-regional interaction. Data from the origin and destination points could be transformed into interactions between the regions (such as distance) if we did not deal with them as mass terms. Traffic was generated by purposefully looking for features, such as business, shopping, healthcare, and education [81]. To exemplify, if someone came to work or traveled to a destination with a particular function, such as to seek out a hospital, theater, or court, they were more likely to endure difficulties related to distance and cost than if they visited places with little function to them. This demonstrated that functional demands influenced factors that resisted physical constraints, which therefore meant that these terms had to be included in the urban human mobility prediction model. In this study, we used this functional distance concept to express functional interaction among the different regions.

**Fig 7:**
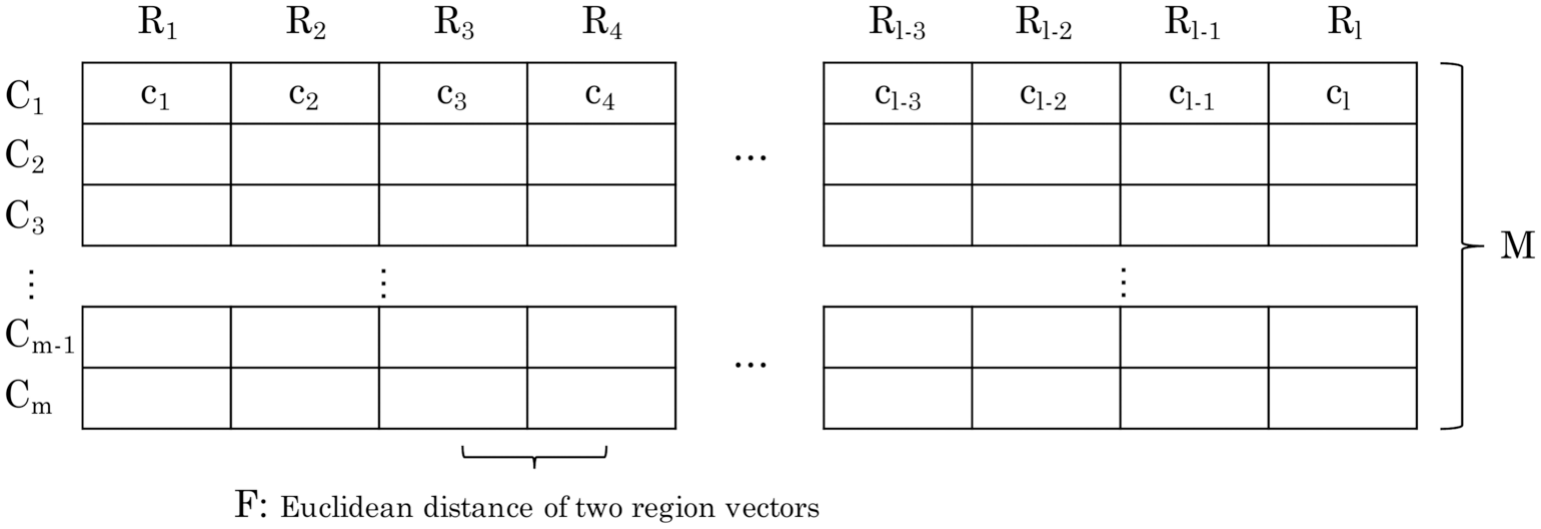
The Concept of the functional distance.

Brown and Horton proposed a functional distance term that could be used to reflect the functional differences between two nodes [1]. Fig 7 shows the basic concept of the functional distance. If we defined a characteristic vector with the characteristic *c* of all regions as *C*, we could make a matrix *M* by combining all characteristic vectors as columns. A row vector of *M* indicated the characteristics of a region, and a region vector was denoted as *R*. Therefore, it was possible to calculate the functional distance from this matrix *M*. When we calculated the functional distance between region *i* and *j*, the functional distance *F*_*ij*_ was the Euclidean distance between two region vectors *R*_*i*_ and *R*_*j*_ (Eq(9)).

$$\begin{array}{c} F_{ij}=\sqrt{\left(R_{i}-Rj\right)\cdot \left(R_{i}-Rj\right)} \end{array}$$ (9)

### Functional distance using public record data

We could make a matrix *M*^*p*^ which contained public record data vectors as characteristics vectors. However, when we calculated the functional distance using the public record data, we saw that it was risky to use matrix *M*^*p*^ with numerous unverified characteristics. First of all, getting a normalized characteristic vector *C*′ that had a range from -1 to 1 by normalizing the vector *C* is required. This had to be done because all characteristics had different values ranges. We called this normalized matrix, *M*^*p*′^. Moreover, the characteristic vectors were similar and duplicated in many cases. For example, since the correlation between total population, male population, and female population was very high, it was difficult to conclude that they described different points, even though they were three distinct features. Therefore, it was necessary to remove duplicated or similar features because these datasets overestimated the descriptive capacity of particular features. The simplest solution to this problem was to eliminate overlapping features by using the common dimensionality reduction technique known as Principal Components Analysis (PCA).

PCA is a dimensionality reduction technique based on orthogonal linear transformation. It decomposes original samples by establishing the first principal component when the entire data on the position of the axis contain the greatest variance. Then, the size of the variance determines the following principal components. In our case, it was possible to reduce the number of dimensions of vectors that were derived from the public records by determining the principal components that could distinguish various types of data equally well. This was particularly useful for our purposes, since dimensionality reduction and weight adjustment could solve the problem of distortion in our description capacities caused by similar and duplicated data, thus requiring no manual analysis of the modified and added data in order to calculate the functional distance between regions. Based on this, matrix *M*^*p*′′^ was obtained by conducting PCA on the matrix *M*^*p*′^ that contained the normalized public record vectors of the regions. *M*^*p*′′^ contained many principal component vectors, but the top *n* principal components were used only to decrease the number of dimensions, therefore making it possible to remove the effects of similar and duplicated data. Finally, the functional distance with the public record data and PCA ($F_{ijn}^{p}$) denoted the Euclidean distance between two region vectors $R_{i}^{p}$ and $R_{j}^{p}$, and was determined from *i* and *j* by using the top *n* principal components in the matrix *M*^*p*′′^.

**Fig 8:**
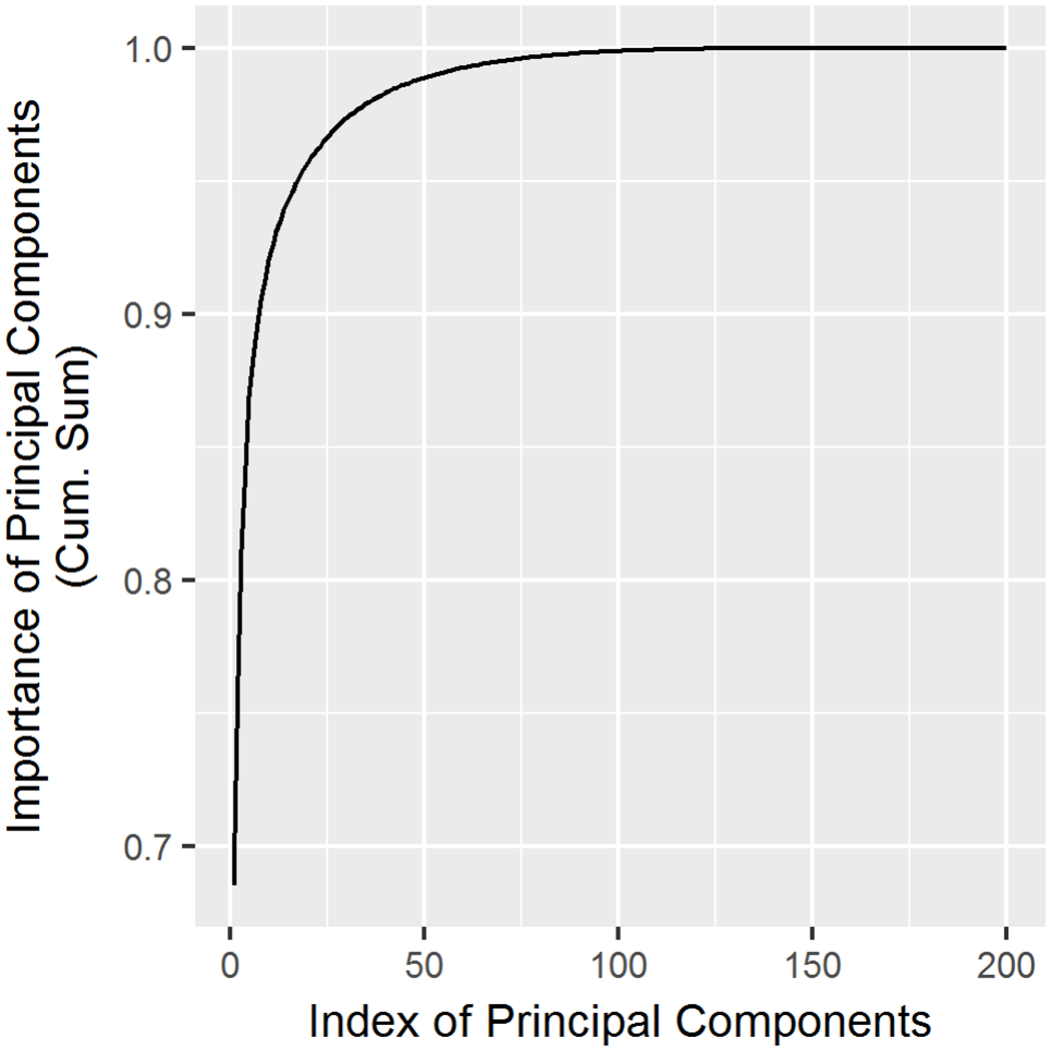
Importance cumulative sum of principal components.

It was necessary to consider the importance of the components used to determine *n*, as this indicated the explanatory power of the original data. If the importance of a value was higher, it reflected the original data well. Of course, if we used all the components, it would have been possible to reflect the original data perfectly. However, since the goal of using PCA was to reduce the number of dimensions, we wanted to obtain enough explanatory power with a relatively small number of components. When considering the cumulative volume in order of its importance from Fig 8, the top component covered 69%, whereas the top three components were needed for 80% coverage. We used the top eight components to cover 90% of the original data, which corresponded to 2% of the actual dataset. In other words, we covered enough of the original data with a small number of components and provided benefits for calculation via reduction of the dataset’s dimensions.

### Functional distance using SNS data

**Fig 9:**
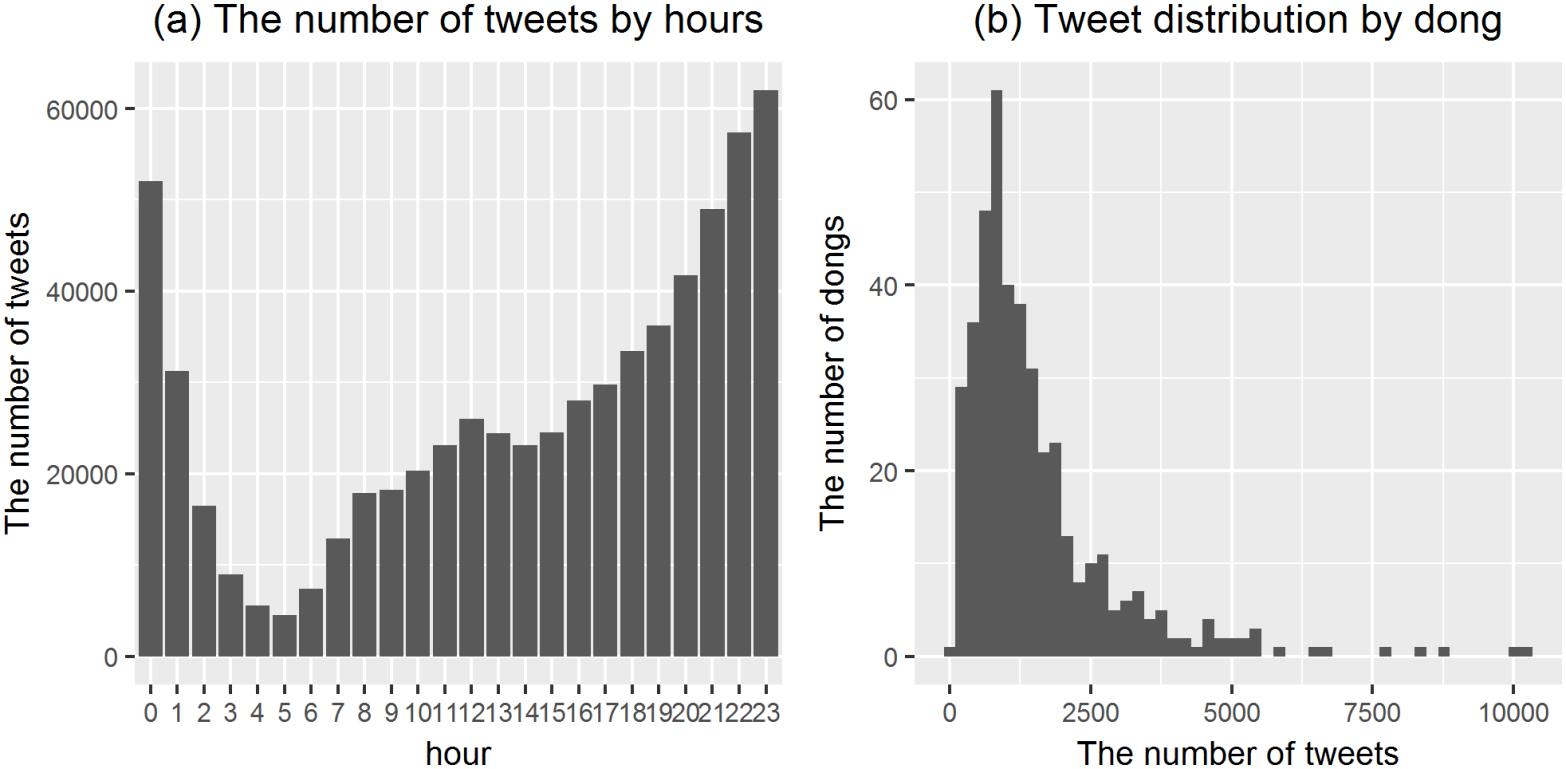
Temporal and spatial distributions of tweets. (a) The number of tweets by hours (b) Tweet Distribution by dong.

When we looked at the basic information of our tweet data, the most number of tweets was generated between the hours of 10 p.m. and 11 p.m., whereas the least was generated between 4 a.m. and 5 a.m. (Fig 9a). Considering that many people tended to stay home at late night, it could be assumed that the contents of their tweets reflected their daily lives. Fig 9b shows the number of tweets was concentrated in several regions, including Gangnam, Gwanghwamun, and Yeouido. These are known as central business districts, and represented tweets from many of dongs. This was similar to traffic distribution, even though it was less skewed. This was a natural phenomenon because many tweets were generated in a place where many people gather.

To understand the functions of urban regions from tweet contents, we focused on the topic of the tweets’ texts. First of all, we separated tweet text through a morpheme analyzer, found relationships among morphemes through the word2vec module, and built word clusters using a *k*-means clustering algorithm.

When we used *k*-means clustering, finding cluster number *k* was necessary in order to get a better result. To do this, we used the score that represented the average distance from the centers of clusters to all points in their cluster. We selected 20 as the number *k* because changes in the score were sharply reduced. Table 5 shows the major topics, words, and the ratio of 20 clusters as results of the *k*-means clustering algorithm. Even though each cluster did not consist of perfectly distinguishable categories, it was still possible to define the major topic because clusters consisted of words with similar contents.

**Table 5:** Major topics, words and ratio of clusters.

Cluster #	Major topics and words	Ratio (%)
0	Schedule, time, season	3.29
1	Bad feelings	6.96
2	Success, effort, experience, attitude	9.18
3	Korea, America, interview, press	2.43
4	Cheer up, happy, greetings	2.68
5	Body parts, physical condition, Glasses/Shoes	6.36
6	Culture, musical, music, broadcast, performance	2.96
7	Food, drink	2.76
8	Transportation (mode, place name)	3.66
9	Family, friend, pet, personal relationship	4.96
10	Politics, politicians, parliament, parties	3.33
11	religion, spirit, wish	4.21
12	Usual, nowadays, current feelings	9.36
13	Day of the week, commuting, time	5.39
14	The name of administrative district	3.34
15	Famous places, shopping	4.29
16	Celebrities’ Twitter ID	3.60
17	Exclamation, emoticon	11.05
18	Love, happiness, comfort	5.64
19	Company name, event, advertising	4.56

When the context of clusters was considered, there were clusters about current status and feelings (1, 2, 4, 12, and 18), daily lives (0, 8, 9, and 13), exclamation and emoticon (17), culture (6), food and drink (7), famous places and shopping (15), politics and social issues (3, 10), fashion, beauty and health topics (5), religion (11), and advertising (19). When we examined the overall ratio, we found many everyday words rather than words that were related to special events. Therefore, it was possible to argue that the tweets reflected the daily lives of users in urban regions.

The matrix *M*^*s*^, which contained the word count vectors, was defined as a characteristics vector. The word count vector contained the word count of each cluster in order to calculate the functional distance using SNS data. After that, normalization of the word number was needed because the total number of words in each vector was different. The normalized matrix *M*^*s*′^ was obtained from this process. Finally, the functional distance with SNS data $F_{ijk}^{s}$ was defined as the Euclidean distance between two normalized vectors $R_{i}^{s}$ and $R_{j}^{s}$ from regions *i* and *j* with *k* number of word clusters. In the next section, we tested the modified gravity models using two different functional distances in order to figure out if they could improve the traffic prediction in the city.

## Modified gravity models using the calculated functional distance

Table 6 (1)-(2) show that significant positive correlations existed between the two different functional distances and the traffic volume determined as a result of regression analysis of the modified gravity model. In other words, distance and mass conditions were controlled, which meant that the farther the functional distance was, the greater the traffic volume. This indicated that the functional distance between regions could be used as another variable for predicting urban traffic volume.

**Table 6:** Regression results of functional distance models with ZINBPML estimator.

	*Dependent variable:*		
	Traffic		
	(1)	(2)	(3)
Origin residential population (log)	−0.091***	−0.100***	−0.090***
	(0.011)	(0.011)	(0.011)
Destination residential population (log)	0.032***	−0.003	0.035***
	(0.007)	(0.007)	(0.007)
Origin employee (log)	0.241***	0.295***	0.241***
	(0.007)	(0.007)	(0.007)
Destination employee (log)	0.187***	0.235***	0.187***
	(0.005)	(0.005)	(0.005)
Origin tweet (log)	0.779***	0.820***	0.780***
	(0.007)	(0.007)	(0.007)
Destination tweet (log)	0.658***	0.709***	0.659***
	(0.005)	(0.005)	(0.005)
Time (log)	−2.249***	−2.203***	−2.250***
	(0.008)	(0.008)	(0.008)
*F*^*p*^ (log)	0.464***		0.462***
	(0.008)		(0.008)
*F*^*s*^ (log)		0.056***	0.028***
		(0.008)	(0.008)
Constant	8.547***	6.311***	8.600***
	(0.149)	(0.145)	(0.150)
Observations	178,929	178,929	178,929
Log Likelihood	−904,576.800	−906,056.900	−904,571.100

*p < 0.1

**p < 0.05

***p < 0.01

Table 6 is a model that contains both functional distance values so as to determine the impact they have. Even though the functional distance calculated using public records and SNS data were in a model, their significance and the sign of their coefficients did not change. This meant that both functional distance terms were significant and independent terms that could be used to predict human mobility in the city.

Since all of the results above had meaning when the mass and distance terms were controlled, it was difficult to argue that functional distance terms were decidedly better than other terms in the modified gravity model. Mass values had significant, positive coefficients, whereas distance values had significant, negative coefficients in both the original and modified gravity models. Nevertheless, when comparing the log likelihood values, modified models with functional distance terms were able to predict traffic flow better than existing models. Therefore, we maintain that the functional distance we had previously defined could be used to improve traffic prediction in the city. Moreover, we conducted a log likelihood ratio test between models with functional distance terms and those without functional distance terms. Based on results from this test, we could ensure that models with functional distance had better performance than existing models.

We already know that human movements are not random, but rather, purposeful [4, 44]. This means that traffic prediction models should consider the social features of people. We focused on the functions of urban reasons in order to reflect the social features extrapolated using public records and SNS data. Even though SNS data, including tweets, contained both the aforementioned social features and their mobility patterns, existing studies only focused on the mobility patterns rather than contents [40, 41]. Finding contextual features from SNS data was one important contribution of this work.

Traffic is the relationship between two regions, therefore, variables that could reflect the social relationship between two regions were required. For this reason, we did not use those features at the node level, but at the link level. Our results indicated that the functional distance related to social differences among regions was a significant variable for human mobility prediction in the city.

## Conclusion

In this study, we verified the best-use terms for a gravity model to determine urban human mobility. With the exception of the floating population (which has a tautological problem), the number of tweets was found to be the best value for predicting urban human mobility for the mass term. However, all mass values were used at the same time because all mass values were considered to be significant even if their explanatory power values were different.

For the distance values, the straight-line distance often used in existing gravity models does not reflect the complex structure of the urban transportation system. We were able to show that time and travel-distance were possible variables that could be used to replace the straight-line distance value. We discovered that time seemed to be a better fit relative to the other values because the time variable contained more context obtained from the structure of the transportation system.

Finally, we verified that the functional distance had a significant relationship with traffic flows in the city using the public record and SNS data. Therefore, it was possible to use the features of urban regions for urban human mobility prediction regardless of the types and amount of data with PCA and ESF.

Even though this study revealed urban human mobility patterns more clearly, but there are additional issues that need to be addressed beyond the scope of this work. It is necessary to apply our approach to long-term human mobility patterns in the city. We had focused on the characteristics of daily movement patterns because of data access limitations, but it is expected that long-term pattern analysis would yield additional factors that short-term analysis cannot. If it is possible to predict the period when functional changes of the regions affect human behavior in the city, we can predict the traffic patterns caused by it.

In conclusion, the most significant contribution of this study was the integration of physical and social factors into a human mobility prediction model. The functional distance was a way to consider the social factors of urban regions. By adding this term to the human mobility prediction model, we proved that social difference was a significant reason for the movement of people through a city. Physical factors, including population and distance, could be used to explain much about human mobility. However, it was necessary to incorporate the huge, continuously collected volumes of data in order to predict traffic with an accuracy that physical factors alone simply could not account for. We anticipate that this approach can be utilized to improve practices in areas, such as urban and transportation planning.

## Supporting information

S1 File Traffic data. Traffic volume data for basic administrative districts in Seoul from April 12 to April 14, 2013.
(ZIP)

S2 File Public record data. Public record data for Seoul, 2013.
(ZIP)

S3 File SNS data. Tweets created in Seoul, 2013.
(ZIP)
